# Chemical Composition of Tomato Seed Flours, and Their Radical Scavenging, Anti-Inflammatory and Gut Microbiota Modulating Properties

**DOI:** 10.3390/molecules26051478

**Published:** 2021-03-09

**Authors:** Uyory Choe, Jianghao Sun, Elena Bailoni, Pei Chen, Yanfang Li, Boyan Gao, Thomas T. Y. Wang, Jiajia Rao, Liangli (Lucy) Yu

**Affiliations:** 1 Department of Nutrition and Food Science, University of Maryland, College Park, MD 20742, USA; uyory.choe@ndsu.edu (U.C.); ebailoni@terpmail.umd.edu (E.B.); gaoboyan@sjtu.edu.cn (B.G.); lyu5@umd.edu (L.Y.); 2 Food Ingredients and Biopolymers Laboratory, Department of Plant Sciences, North Dakota State University, Fargo, ND 58102, USA; jiajia.rao@ndsu.edu; 3 Methods and Application of Food Composition Laboratory, Beltsville Human Nutrition Research Center, Agricultural Research Service, United States Department of Agriculture, Beltsville, MD 20705, USA; Jianghao.Sun@ars.usda.gov (J.S.); Pei.Chen@ars.usda.gov (P.C.); 4 Institute of Food and Nutraceutical Science, School of Agriculture and Biology, Shanghai Jiao Tong University, Shanghai 200240, China; 5 Diet, Genomics and Immunology Laboratory, Beltsville Human Nutrition Research Center, Agricultural Research Service, United States Department of Agriculture, Beltsville, MD 20705, USA; Tom.Wang@ars.usda.gov

**Keywords:** tomato seed, gut microbiota, polyphenol, antioxidant, anti-inflammation

## Abstract

In the current study, the chemical composition and total phenolic content of tomato seed flours, along with potential health beneficial properties, including free radical scavenging capacities, anti-inflammatory capacities, and gut microbiota profile modulation, were examined using two different batches. Eight compounds were identified in the tomato seed flour, including malic acid, 2-hydroxyadipic acid, salicylic acid, naringin, *N*-acetyl-tryptophan, quercetin-di-*O*-hexoside, kaempferol-di-*O*-hexoside, and azelaic acid. The total phenolic contents of tomato seed flour were 1.97–2.00 mg gallic acid equivalents/g. Oxygen radical absorbing capacities (ORAC), 2,2-diphenyl-1-picrylhydrazyl radical scavenging capacities (DPPH), and 2,2′-azino-bis(3-ethylbenzothiazoline-6-sulfonic acid) cation radical scavenging capacities (ABTS) were 86.32–88.57, 3.57–3.81, and 3.39–3.58 µmoles Trolox equivalents/g, respectively, on a per flour dry weight basis. The mRNA expression of the pro-inflammatory markers, interleukin-1 beta (IL-1β), interleukin-6 (IL-6), and tumor necrosis factor alpha (TNF-α), were dose-dependently suppressed by tomato seed flour extracts. The extracts altered five of the eight bacterial phyla and genera evaluated. The results may provide some scientific support for the use of tomato seed flour as value-added food ingredients.

## 1. Introduction

**Figure 1:**
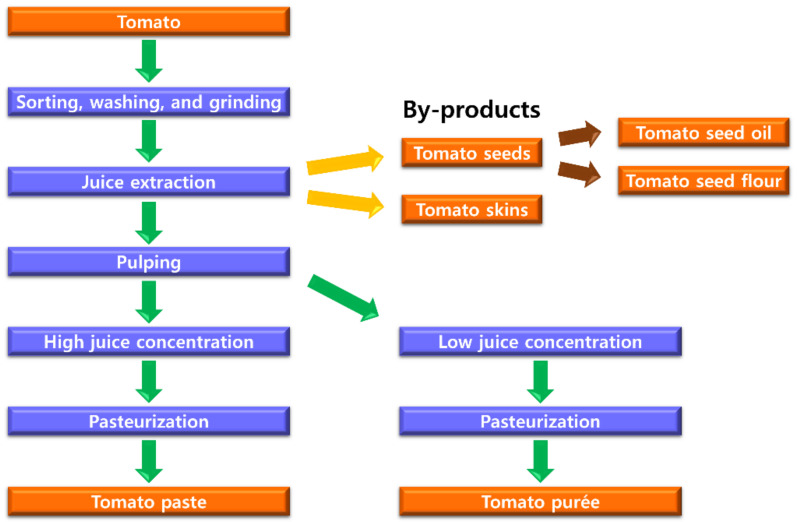
Tomato purée and paste processing scheme. Tomato seeds can be further processed into tomato seed oil and flour.

Tomato is the world’s second most-consumed vegetable and it accounts for 19% of all vegetable consumption [1]. Tomato is consumed raw, stuffed, grilled, or pickled. However, tomato is more often consumed as processed food products. Among a number of processed tomato products, major products are tomato purée and paste [2]. Tomato purée and paste both serve as the main ingredient for tomato-based food products, such as pasta sauce, pizza sauce, salsas, ketchup, dipping sauce, soups, drinks, and frozen foods. In 2017 and 2018, 35.7 million metric tons of tomatoes were processed by the world’s top 40 companies [2] into mostly tomato purée and paste, since these products are widely used food ingredients. Tomato skins and seeds are removed during tomato purée and paste processing (Figure 1). These wastes are increasing the final disposal costs to the industry and causing environmental contaminations. Developing potential utilization of tomato by-products may reduce the waste and minimize the cost.

In recent years, the use of natural food by-products has been increasing alongside increasing interest in sustainability. This effort can reduce environmental contamination and, at the same time, may add value to the final food products, since natural food by-products are often rich in bioactive compounds. For example, the tomato seed oil is rich in lycopene and tocopherols [3] and tomato skin is rich in essential amino acids and polyphenolic compounds [4]. In addition, tomato seed oil and skin have been evaluated for their potential health beneficial properties. In 2011, Elbadrawy and Sello reported the antioxidant activities of tomato skin extracts [4], whereas Shao and others reported tomato by-products, such as tomato skin, seed oil, and defatted seed for their potentials in lowering plasma and liver cholesterol levels [5]. Because of bioactive content and health beneficial properties, tomato skin and seed oil are currently utilized in salad topping and dressing, respectively. However, other than hypocholesterolemic properties [5], tomato seed flour may possess more health beneficial properties that were worth further study. In addition, there is a little information on the bioactive components that are responsible for the bioactivities in tomato seed flour, warranting additional research.

In this study, two batches of tomato seed flours were extracted and analyzed for their chemical compositions, total phenolic contents, and potential health benefits, particularly free radical scavenging capacities, anti-inflammatory capacities, and gut microbiota profile modulation. The findings could serve as a scientific basis for the development of food products using tomato seed flours to improve human health, as well as further investigation of the biological benefits and molecular mechanisms that are behind it.

## 2. Results and Discussion

### 2.1. Chemical Composition of the Tomato Seed Flour Extracts

**Figure 2:**
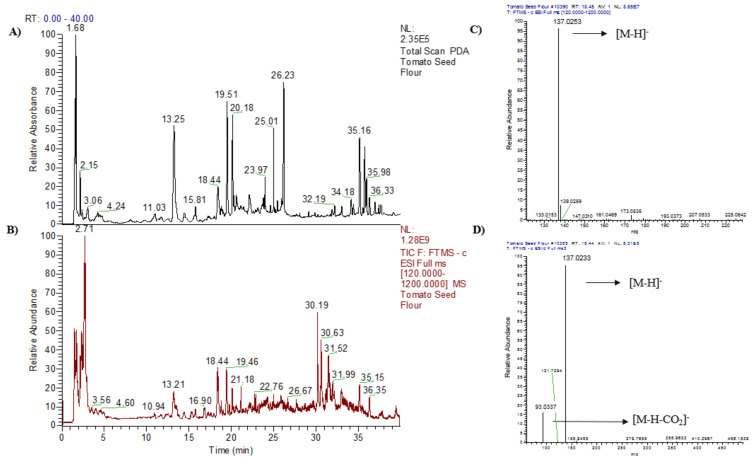
(**A**) Typical Ultra-High-Performance Liquid Chromatography-PDA (UHPLC-PDA) chromatogram and (**B**) total ion current (TIC) chromatogram of tomato seed flour extracts; (**C**) MS; and, (**D**) MS/MS spectra of peak 3 at the retention time of 18.44 min.

In both tomato seed flour extracts, named TSF1 and TSF2, a total of eight compounds, namely malic acid, 2-hydroxyadipic acid, salicylic acid, naringin, *N*-acetyl-tryptophan, quercetin-di-*O*-hexoside, kaempferol-di-*O*-hexoside, and azelaic acid, were tentatively identified (Table 1 and Figure 2).

The tentative identification of the eight peaks were based on the theoretical, experimental molecular ions ([M−H]^−^) and the major MS/MS fragment ions, along with the MS data in the literatures. For example, peak 3 had a *m/z* [M–H]^−^ of 137.0233, which refers to the formula, C_7_H_6_O_3_ (mass error, −0.72 mmμ) (Figure 2C). The MS/MS spectrum showed a *m/z* of 137.0233, which was a parent ion and the fragmental ion had a *m/z* 93.0337, which was resulted from the elimination of a carboxyl group from the molecular ion (Figure 2D). This fragmentation matched with that previously reported for salicylic acid by Chhonker and Pastor’s groups [6,7]. Taking together, peak 3 was tentatively identified as salicylic acid. Similarly, peak 1 of the tomato seed flour extracts showed a *m/z* [M − H]^−^ of 133.0135, which corresponds to the formula of C_4_H_6_O_5_ (mass error, −0.75 mmμ) (Table 1). The peak 1 had the fragment ion at *m/z* of 71.0131 (Table 1). These *m/z* values were in line with the previously reported malic acid *m/z* values proposed by Kadhi and others [8], and peak 1 was therefore tentatively identified as malic acid. The same approach was applied to identify the other compounds found in tomato seed flour extracts. As a result, 2-hydroxyadipic acid [9], naringin [10,11], *N*-acetyl-tryptophan [12,13,14], quercetin-di-*O*-hexoside [15,16], kaempferol-di-*O*-hexoside [16], and azelaic acid [17,18] were tentatively identified.

Among the eight identified compounds, four compounds, including salicylic acid, naringin, quercetin-di-*O*-hexoside, and kaempferol-di-*O*-hexoside, were polyphenolic compounds (Figure S1). Polyphenolic compounds are known to possess potential health beneficial properties, such as reducing the risk of arthritis [19], cancers [20], diabetes [21], obesity [22], and cardiovascular diseases [23].

Salicylic acid is a phenolic acid that is commonly found in plants. It is widely used in medicines treating skin redness. In addition, salicylic acid is also a metabolite of aspirin, acetylsalicylic acid, a pharmaceutical compound. It has been reported that salicylic acid is responsible for the anti-inflammatory acts of aspirin [24]. Naringin, quercetin-di-*O*-hexoside, and kaempferol-di-*O*-hexoside are polyphenol glycosides. In many cases, polyphenols that are found in plants exist as glycosides [25]. In polyphenol glycosides, the sugar moiety is often hydrolyzed by the digestive enzymes or gut microflora to release the polyphenol aglycone(s) [26]. Therefore, it is important to understand and evaluate both intact and hydrolyzed forms of polyphenols for potential health beneficial properties. For example, naringin is a flavonoid and polyphenol glycoside found in fruits, vegetables, and herbs, and it may be hydrolyzed to naringenin, the aglycone of naringin in vivo. Previously, Cavia-Saiz and others evaluated the antioxidant properties of both naringin and naringenin [27]. They found that naringenin and naringin both showed antioxidant capacities, but, when compared to naringin, naringenin showed a greater antioxidant capacity. In addition, it has been reported that naringenin possessed cholesterol-lowering capacity [28]. Similarly, quercetin-di-*O*-hexoside and kaempferol-di-*O*-hexoside may both be hydrolyzed to their aglycones during digestion. Quercetin is a flavonoid and has a number of health beneficial properties, including free radical scavenging capacities, anti-tumor activities, and reducing the risk of cardiovascular and neurodegenerative diseases [29]. Kaempferol is also a flavonoid that is found in plant food sources, such as kale, spinach, and broccoli. Kaempferol has been a widely investigated polyphenolic compound because of its anti-cancer activity [30]. It has been reported that the anti-cancer activity of kaempferol is mainly by modulating cell signals that are related to the programmed cell death, new blood vessel formation, and the spread of cancer cells. These possible in vivo health beneficial properties of hydrolyzed polyphenols are important. However, it is also important to note that the glycoside forms of polyphenolics may contribute potential health beneficial properties, such as free radical scavenging capacities, anti-inflammatory capacities, and gut microbiota modulation. For instance, a previous study found that glycoside forms of kaempferols, including kaempferol-7-*O*-glucoside, kaempferol-3-*O*-rhamnoside, and kaempferol-3-*O*-rutinoside, showed anti-proliferative capacities, free radical scavenging capacities, and anti-inflammatory capacities [31]. Furthermore, other studies suggest that glycoside forms of kaempferol, quercetin, and naringin are subjects to possible hydrolysis by gut microbiota and they may contribute to gut health [32].

In addition, four non-phenolic compounds were found in the tomato seed flour extracts. These compounds are malic acid, 2-hydroxyadipic acid, *N*-acetyl-tryptophan, and azelaic acid (Table 1 and Figure S1). Malic acid is an organic acid that is widely found in fruits and vegetables and contributes sour taste. Besides, malic acid can act as a bioavailability enhancer of minerals such as iron [33]. Tryptophan is an essential amino acid and involved in human health conditions including kidney diseases, cardiovascular diseases, diabetes, depression, sleep, and social behavior [34]. In the current study, tryptophan was identified as an acetyl form. However, previous studies suggested that *N*-acetyl-tryptophan can also act as a tryptophan through the metabolism [35,36]. Azelaic acid is a carboxylic acid that is widely found in grains, such as rye, oat, barley, wheat and sorghum [37]. Azelaic acid has been reported to possess protective effects against oxidative stress in several organs [37]. Together, tomato seed flour has the potential for its utilization as a food additive or a functional food ingredient.

### 2.2. Total Phenolic Content and Free Radical Scavenging Capacities of Tomato Seed Flour Extracts

Total phenolic content (TPC) is often closely connected to the free radical scavenging capacity and other potential health beneficial properties. In the current study, two different batches of tomato seed flour extracts, TSF1 and TSF2, showed TPC values of 2.00 and 1.97 mg gallic acid equivalents/g of dry seed flour (mg GAE/g), respectively (Table 2). Previously, Toor and Savage evaluated the TPC of tomato seeds while using three different tomato cultivars, including *Excell*, *Tradiro* and *Flavourine*, and found TPC values of 0.35, 0.29, and 0.21 mg GAE/g fresh weight, respectively [38]. When compared to Toor and Savage’s TPC values, TSF1 and TSF2 showed greater TPC values under the experimental conditions. This may be due to the different samples and extraction methods. In the present study, 50% acetone was used to extract the tomato seed flour, where Toor and Savage used whole tomato seeds with 100% hexane and a combination of acetone, water, and acetic acid (70:29.5:0.5, *v/v/v*) as solvents for lipophilic and hydrophilic compounds, respectively [38]. In 2002, Kaur and Kapoor evaluated the TPC of tomato fruit and found a TPC value of 0.68 mg GAE/g [39]. TSF1 and TSF2 had greater TPC values when compared to tomato fruits (Table 2).

TSF1 and TSF2 showed oxygen radical absorbing capacities (ORAC), 2,2-diphenyl-1-picrylhydrazyl radical scavenging capacities (DPPH) and 2,2′-azino-bis(3-ethylbenzothiazoline-6-sulfonic acid) cation radical scavenging capacities (ABTS) of 88.57–86.32, 3.57–3.81, 3.39–3.58 µmoles Trolox equivalents/g of seed flour (µmoles TE/g), respectively (Table 2). ORAC values were much greater when compared to DPPH or ABTS. These differences may be due to the different structure of the radicals and reactivities between the radical and polyphenolic compounds found in tomato seed flour extracts. During the reaction of free radical scavenging by antioxidants, radicals are quenched by two mechanisms in most cases, namely hydrogen atom transferring (HAT) or single electron transferring (SET) [40]. The mechanisms of ABTS and DPPH assays are based on mixed HAT and SET; however, the ORAC’s mechanism is solely based on HAT. Accordingly, the antioxidant capacity might be different based on different assays; therefore, two or more assays are needed to evaluate a selected antioxidant compound.

Previously, Zhou and Yu evaluated the free radical scavenging capacities of ten tomatoes that were grown in Colorado and found ABTS values in the range of 5.4–20.9 µmoles TE/g dry weight [41]. They also reported TPC values of ten tomatoes. TPC values were in the range of 2.9–5.0 mg GAE/g [41]. When compared to the current study’s TPC and ABTS values, both of the values were greater for tomatoes grown in Colorado. These results suggest a possible correlation between the TPC and free radical scavenging capacities, which was also confirmed by the Pearson correlation analyses in this study. As a result, TPC showed a significantly positive correlation with ORAC (r = 0.975, *p* ≤ 0.01), DPPH (r = 0.991, *p* ≤ 0.01), and ABTS (r = 0.987, *p* ≤ 0.01) values, which suggested that total phenolics in tomato seed may directly contribute to the radical scavenging capacities. In 2018, Perea-Domı’nguez and others tested free radical scavenging capacities of two tomato varieties, Grape and Saladette, and found ORAC and DPPH values of 1631.39–2426.53, 307.8–465.17 µmoles TE/g dry weight, respectively [42]. Overall, tomato seed flours had lower free radical scavenging capacities on a per dry weight basis as compared to tomato fruits.

### 2.3. Anti-Inflammatory Capacities of Tomato Seed Flour Extracts

**Figure 3:**
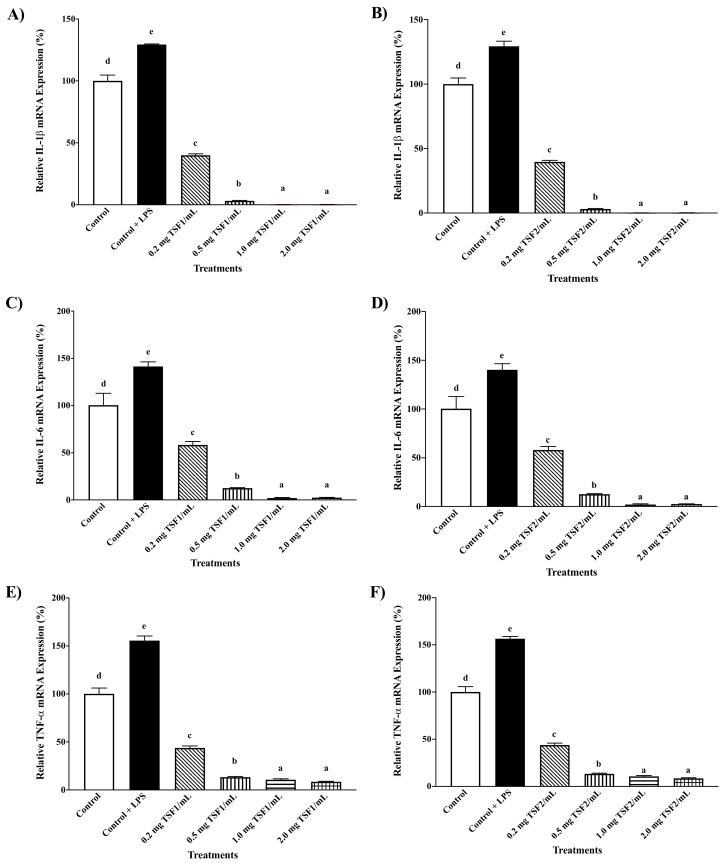
Anti-inflammatory capacities of tomato seed flour extracts at concentrations of 0.2, 0.5, 1.0, and 2.0 mg tomato seed flour (TSF)/mL in THP-1 macrophages, (**A**,**C**,**E**): TSF1, the first batch of tomato seed flour extract and (**B**,**D**,**F**): TSF2, the second batch of tomato seed flour extract. Control, No-treatment; LPS, lipopolysaccharides; IL-1β, interleukin-1 beta; IL-6, interleukin-6; TNF-α, tumor necrosis factor alpha; Different letters demonstrate significant difference (*p* ≤ 0.05).

TSF1 and TSF2 both demonstrated dose-dependent anti-inflammatory capacities against pro-inflammatory markers, including interleukin-1 beta (IL-1β), interleukin-6 (IL-6), and tumor necrosis factor-alpha (TNF-α) (Figure 3). The treatments with different concentrations of tomato seed flour extracts at 0.2, 0.5, 1.0, and 2.0 mg tomato seed flour (TSF)/mL were capable of inhibiting the lipopolysaccharide (LPS) stimulated IL-1β mRNA expression by 69, 97, 99, 99, 69, 97, 99, and 99%, respectively (Figure 3). Similarly, TSF1 and TSF2 dose dependently inhibited IL-6 and TNF-α mRNA expressions under the experimental conditions (Figure 3). It needs to be pointed out that all of the tomato seed flour extracts with different concentrations used in this test had no adverse effects on THP-1 macrophages viability according to MTT assay (Figure S2).

Navarrete and others previously reported the anti-inflammatory capacities of tomato while using macrophages that were differentiated from THP-1 monocytes and LPS as a stimulant on the expression of proinflammatory cytokines [43]. The tomato extract was able to inhibit the gene expressions of the pro-inflammatory cytokines, including IL-1β and TNF-α. However, the anti-inflammatory capacities of tomato seed flour have not been evaluated in the past. Hence, this is the first report of a potential anti-inflammatory effect for tomato seed flours.

The inflammation process is often closely related to free radicals and chronic diseases. The former, free radicals are produced by a many different sources within the human body. Sources are often divided into two groups, endogenous and exogenous sources. Endogenous sources are mitochondria, peroxisomes, and phagocytes, while exogenous sources are cigarette smoking, air pollution, radiation, some medications, and ozone [44]. A primary cause of chronic diseases is oxidative stress, an imbalance between antioxidants, and free radicals. In the human body, there is a transcription factor, named nuclear factor erythroid-derived 2-like 2 (NRF2), which modulates the production of antioxidants and detoxifying products. These products can protect the damage that is caused by oxidative stress. However, this may not be enough to protect the body from oxidative stress, and the antioxidants from foods are important for overall antioxidative status in the human body.

When oxidative stress is induced, immune cells start to react. Among many immune cells, macrophages play a central role in inflammation and they start to secret pro-inflammatory cytokines and tumor necrosis factors, such as IL-1β, IL-6, and TNF-α. Bioactive compounds in foods can reduce or inhibit the development of these pro-inflammatory cytokines and tumor necrosis factor. For example, kaempferol can act as a nuclear factor kappa-light-chain-enhancer of activated B cells (NF-κB) inhibitor by binding its pathway signaling protein [45]. Similarly, a previous study reported that quercetin inhibited cytokine and inducible nitric oxide synthase expressions through inhibiting the NF-κB pathway [46]. Moreover, naringenin, the aglycone of naringin, has been reported to inhibit enzymes that are responsible for pro-inflammatory responses, such as cyclooxygenase-1 (COX-1) and cyclooxygenase-2 (COX-2) [47]. The result from the present study and the anti-inflammatory capacities of polyphenolic compounds reported by previous studies suggest that tomato seed flour has potential anti-inflammatory capacities, and it may be utilized in food additives or functional foods for improving human health.

### 2.4. Gut Microbiota Profile Modulation of Tomato Seed Flour Extracts

Research on gut microbiota has gained growing attention in recent years, owing to their notable functions in the human body. The gut microbiota profile has been shown to be correlated with host health conditions, starting from metabolic diseases to digestive diseases and colon cancer [48]. Maintaining a healthy gut microbiota profile is a key to maintaining a good state of health. However, each organism has a different gut microbiota profile and, in addition, along with the aging process, the gut microbiota profile changes. For example, a naturally delivered baby and a cesarean delivered baby may have different gut microbiota profiles. Additionally, each individual has a different metabolism rate. Therefore, the speed of the aging process may be distinct. These intrinsic factors are not changeable. On the other hand, there are extrinsic factors that can shift the gut microbiota profile. These factors include exercise, stress, antibiotics, and diet. Among these extrinsic factors, the diet seems to have the biggest impact, since food is what human beings eat every day.

**Figure 4:**
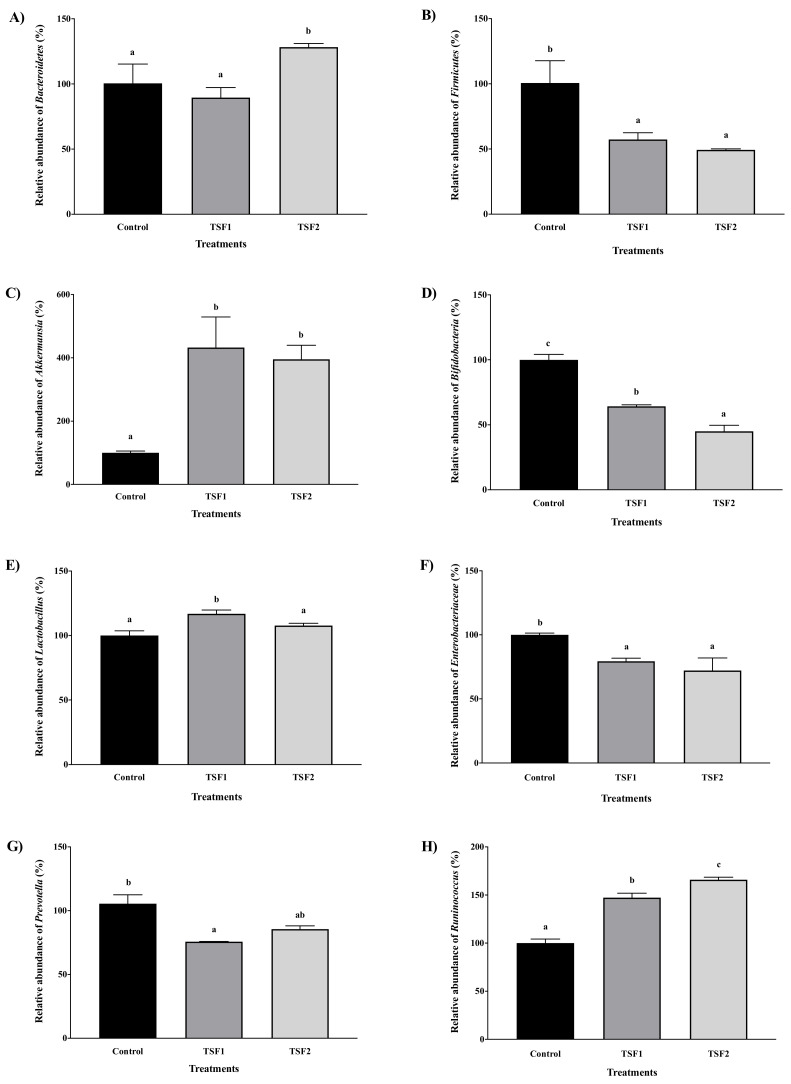
Gut microbiota profile modulation of tomato seed flour extracts, (**A**) *Bacteroidetes* phylum, (**B**) *Firmicutes* phylum, (**C**) *Akkermansia* genus, (**D**) *Bifidobacterium* genus, (**E**) *Lactobacillus* genus, (**F**) *Enterobacteriaceae* genus, (**G**) *Prevotella* genus, and (**H**) *Ruminococcus* genus. Control, No-treatment; TSF1 and TSF2 represent the first and the second batches of tomato seed flour extracts, respectively; different letters demonstrate a significant difference (*p* ≤ 0.05).

In the current study, a total of eight bacterial taxonomic ranks, including *Bacteroidetes* phylum, *Firmicutes* phylum, *Akkermansia* genus, *Bifidobacterium* genus, *Lactobacillus* genus, *Enterobacteriaceae* genus, *Prevotella* genus, and *Ruminococcus* genus, were used to evaluate the gut microbiota profile change by tomato seed flour extracts. Five phyla and genera showed significant changes (*p* ≤ 0.05) (Figure 4). Among the five significantly changed phylum or genus, the abundance of *Akkermansia* and *Ruminococcus* genera were increased (Figure 4C,H) and *Firmicutes* phylum, *Bifidobacterium* genus, and *Enterobacteriaceae* genus were decreased (Figure 4B,D,F). Even though one batch (TSF2) significantly increased *Bacteroidetes* phylum, the other batch (TSF1) did not show any significant effect (Figure 4A). Additionally, the *Lactobacillus* genus was significantly increased by one batch (TSF1), but not by TSF2 (Figure 4E). Together, these results suggest a possible variation between tomato seed samples in their microbiota modulating properties. In vivo study is needed to further confirm the effects of tomato seed flour on *Bacteroidetes* phylum and *Lactobacillus* genus.

Previously, gut microbiota profile modulation by other vegetable seed flours, including broccoli, carrot, and cucumber, has been reported by our group [49]. In that study, the abundance of *Bacteroidetes* phylum was significantly increased by cucumber seed flour extract and decreased by carrot seed flour extract and not changed by broccoli seed flour extract. In the current study, one batch of tomato seed flour extract (TSF2) significantly increased *Bacteroidetes* phylum, but the fold was much lower than that of cucumber seed flour extract. Furthermore, both batches of tomato seed flour extracts decreased the abundance of *Firmicutes* phylum (Figure 4B). This *Firmicutes* phylum decrease has also been observed in the previous study. In the previous study, broccoli, carrot, and cucumber seed flour extracts significantly decreased the abundance of *Firmicutes* [49]. Additionally, a similar trend was observed in the *Enterobacteriaceae* genus. This study and the previous study both observed significant decreases of the *Enterobacteriaceae* genus (Figure 4F). For probiotic bacteria, *Bifidobacterium* and *Lactobacillus* genera, only the *Bifidobacterium* genus was decreased by TSF1 and TSF2 (Figure 4D,E). This was different to the observations in the previous study that all three vegetable seed flour extracts decreased both probiotic bacteria, *Bifidobacterium* and *Lactobacillus* genera [49].

In gut microbiota, *Bacteroidetes* and *Firmicutes* phyla consist of more than 90% population and they play important roles. *Bacteroidetes* can activate lymphocyte T cell to modulate human immune responses and produce butyric acid [50]. In addition, *Bacteroidetes* are participating in the degradation of toxin and carcinogen and bile acid metabolism [51]. *Firmicutes* are closely related to the aging process and they are possibly involved in fatty acid metabolism [52]. *Bifidobacterium* and *Lactobacillus* genera are probiotics and known to reduce infectious diarrhea and pathogen colonization [53]. *Akkermasia* are a mucin degrading bacterium and maintain gut health [54], and they could reduce body fat mass and adipose tissue inflammation and improve glucose homeostasis [55]. *Prevotella* can be used as a biomarker for gut dysbiosis, since the abundance of *Prevotella* genus is associated with diets that are rich in plants and their components, such as carbohydrates and fibers [56]. *Ruminococcus* possess the abilities to breakdown and use a wide range of plant polysaccharides for host health [57]. Because the tomato seed flour extracts significantly increased both *Akkermansia* and *Ruminococcus* genera, tomato seed flour may be used as a functional food for weight control and improving digestion.

## 3. Materials and Methods

### 3.1. Chemicals and Reagents

The tomato seed flours were donated by the Botanic Innovations (Spooner, WI, USA). PMA (phorbol 12-myristate 13-acetate) (product#: P1585), Trolox (6-Hydroxy-2,5,7,8-tetramethylchroman-2-carboxylic acid) (product#: 238813), gallic acid (product#: G7384), Folin-ciocalteu reagent (2N) (product#: F9252), and sodium carbonate (product#: 223530) were acquired from Sigma Aldrich (Saint-Louis, MO, USA). AAPH (2,2′-Azinobis (2-amidinopropane) dihydrochloride) (catalog#: 992–11062) was acquired from Wako Chemicals (Richmond, VA, USA). The materials for the cell culture were purchased from GIBCO (Grand Island, NY, USA). Materials for PCR (polymerase chain reaction) were acquired from either Thermo Fisher Scientific (Fair Lawn, NJ, USA) or Qiagen (Gaithersburg, MD, USA).

### 3.2. Tomato Seed Flour Extract Preparation

Extraction was performed following the previously reported method with a slight modification [58]. Briefly, 10 g of tomato seed flour was mixed with 50 mL of 50% acetone and then vortexed for 1 min. Subsequently, the mixture was sonicated for 1 min and rested for 24 h. This extract was used to assess free radical scavenging capacities, anti-inflammatory capacities, and gut microbiota profile modulation. For chemical composition analysis, 10 g of tomato seed flour was extracted using a Soxhlet extractor with 50 mL of 100% ethanol.

### 3.3. Ultra-High-Performance Liquid Chromatography-High Resolution Mass Spectrometry (UHPLC-HRMS) Analysis

The UHPLC-HRMS (Themo Scientific, Waltham, MA, USA) system consists of an Orbitrap ID-X tribrid mass spectrometer with a Vanquish UHPLC, including a high-pressure binary pump, thermostatting column temperature control compartment, and an HL Diode Array Detector [59]. The separation was carried out on an Agilent RRHD Eclipseplus C_18_ 2.1 × 150 mm 1.8 µm (Agilent, Palo Alto, CA, USA) with an UltraShield pre-column filter (Analytical Scientific Instruments, Richmond, CA, USA) with a flow rate of 0.3 mL·min^−1^. Solution A (0.1% formic acid in water, *v/v*) and solution B (0.1% formic acid in acetonitrile, *v/v*) were used for gradient elution with the following program. The proportion kept at 2% B (*v/v*) at 0–5 min, and then increased to 10% B at 15 min., to 45% B at 25 min, to 90% at 35 min, and this proportion was kept at 90% to 40 min. The post-run time for re-equilibration was 10 min. The UV-vis spectra were recorded at the range of 190–600 nm for the entire run. The column temperature was set at 50 °C and the sample compartment temperature was set at 4 °C. The injection volume was 2 µL. The MS conditions were set, as follows: sheath gas at 50 (arbitrary units), auxiliary gas at 10 (arbitrary units), and sweep gas at 1 (arbitrary units), spray voltage at 3 kV with negative ionization mode, ion transfer tube temperature at 300 °C, vaporizer temperature at 350 °C, and RF lens at 60%. The full scan mass range was from 120 to 1200 *m/z* with a resolution of 60,000, AGC target value of 200,000 in full scan and 10,000 FTMS/MS, and max ion injection time of 50 ms. The most intense ion was selected for the data-dependent scan with normalization collision energy at 35% in HCD. The data were post-processed using the Xcalibur 2.2 software.

### 3.4. Total Phenolic Content

The total phenolic content was evaluated following the laboratory procedure previously reported with a minor modification [58]. Briefly, 3 mL of deionized water, 50 μL of sample, standard, or solvent (blank), and 250 μL of the diluted Folin–Ciocalteu reagent (0.5 N) were added to the test tube and then vortexed for 1 min. After vortexing, 750 μL of 20% (w/v) sodium carbonate was added to trigger the reaction. After 2 h, a wavelength of 765 nm was used to measure the absorbance. The result was stated as milligrams of gallic acid equivalents per gram of the tomato seed flour (mg GAE/g).

### 3.5. Relative 2,2-Diphenyl-1-picrylhydrazyl (DPPH) Radical Scavenging Capacity

The relative DPPH radical scavenging capacity was examined using a previously reported protocol [60]. Using a wavelength of 515 nm, the absorbance was checked every minute and then recorded for 40 min. The Trolox was used to provide a standard curve. For the value unit, micromoles of Trolox equivalents/g of flour (μmoles TE/g) were used.

### 3.6. Oxygen Radical Absorbing Capacity (ORAC)

According to the previously reported protocol, the oxygen radical absorbing capacity (ORAC) was measured [61]. Different concentrations of the Trolox were dissolved in 50% acetone to generate a standard curve. All other reagents were prepared using 75 mM pH 7.4 phosphate buffer. For the detection, wavelengths of 485 and 535 nm were used for the excitation and emission, respectively. The ORAC value was reported as μmoles TE/g of the flour samples.

### 3.7. 2,2′-azinobis (3-ethylbenzothiazoline-6-sulphonic Acid) Diammonium Salt Cation Radical (ABTS^•+^) Scavenging Capacity

The ABTS cation radical scavenging capacity was evaluated using a previously reported protocol [62]. Briefly, ABTS^•+^ working solution was prepared by oxidizing ABTS with manganese oxide and the absorbance was adjusted to 0.700 ± 0.005 at 734 nm. Trolox was used as the antioxidant standard. For the reaction, 1 mL of ABTS^•+^ working solution was mixed with 80 μL of the sample, standard, or solvent. This mixture was vortexed for 30 s. After 90 s, the absorbance value was recorded at 734 nm. The ABTS value was reported as μmoles TE/g of the flour samples.

### 3.8. Anti-Inflammatory Capacity

THP-1 macrophages were used to assess anti-inflammatory capacity. In short, density of 6 × 10^5^ cells/mL THP-1 macrophages were cultured in six-well plates to achieve 80% confluence. Subsequently, macrophages were cultured with/without the tomato seed flour extracts at concentrations of 0.2, 0.5, 1.0, and 2.0 mg tomato seed flour/mL (mg TSF/mL) for 24 h. For the stimulation, 10 ng/mL of lipopolysaccharide (LPS) was used. After four hours of stimulation with LPS, the cells were lysed for RNA isolation. From RNA, cDNA was synthesized and Real-time PCR was performed using TaqMan probe. TATA box binding protein (TBP) was used as a control primer and IL-1β, IL-6, and TNF-α as inflammatory markers.

### 3.9. Gut Microbiota Analysis

A regular chow diet-fed C57BL/6J mouse’s fecal sample was used to prepare gut microbiota, as previously reported [59]. For the bacterial concentration calculation, an OD600 value of 1 = 8 × 10^8^ cells/mL was used. Briefly, 1 × 10^7^ cells/mL of bacterial cells were cultured in M9 broth with/without tomato seed flour extract in 15 mL and 50 mL tubes for 6 h with shaking. After 6 h, the bacterial cells were collected by centrifugation at 5000 rpm for 5 min. The bacterial DNA were extracted using Precellys lysing and QIAamp DNA mini kits. Specific forward and reverse primer sequences used in this study were shown, as follows: *Akkermansia* (Forward: 5′-CAGCACGTGAAGGTGGGGAC-3′, Reverse: 5′-CCTTGCGGTTGGCTTCAGAT-3′); *Bacteroidetes* (Forward: 5′-GGARCATGTGGTTTAATTCGATGAT-3′, Reverse: 5′-AGCTGACGACAACCATGCAG-3′); *Bifidobacterium* (Forward: 5′-TCGCGTCYGGTGTGAAAG-3′, Reverse: 5′-CCACATCCAGCRTCCAC-3′); *Enterobacteriaceae* (Forward: 5′-CATTGACGTTACCCGCAGAAGAAGC-3′, Reverse: 5′-CTCTACGAGACTCAAGCTTGC-3′); *Firmicutes* (Forward: 5′-GGAGYATGTGGTTTAATTCGAAGCA-3′, Reverse: 5′-AGCTGACGACAACCATGCAC-3′); *Lactobacillus* (Forward: 5′-GAGGCAGCAGTAGGGAATCTTC-3′, Reverse: 5′-GGCCAGTTACTACCTCTATCCTTCTTC-3′); *Prevotella* (Forward: 5′-TCCTAGGGAGGCAGCAGT-3′, Reverse: 5′-CAATCGGAGTTCTTCGTG-3′); and *Ruminococcus* (Forward: 5′-GGCGGCCTACTGGGCTTT-3′, Reverse: 5′-CCAGGTGGATAACTTATTGTGTTAA-3′). Real-time PCR was carried with the SYBR probe using a previously reported protocol [59].

## 4. Conclusions

The current study observed several potential health beneficial polyphenolic compounds in tomato seed flour extracts, along with several possible health beneficial properties, including free radical scavenging capacities, anti-inflammatory capacities, and gut microbiota profile modulation. The results might be used to improve human health by increasing the utilization of tomato seed flour as a food additive or functional food ingredient.

## 5. Patents

The current tomato seed flour research has been filed as a Method for Modulating Gut Microbiota (Provisional US Patent application, 63/007734 with a University of Maryland invention disclosure number LS-2018-193).

## Figures and Tables

**Table 1:** The characterization of compounds present in tomato seed flour extracts.

ID	Rt (min)	Exptl. [M–H]^−^	Fragment Ions	Formula	Mass Error (mmμ)	Tentative Identification	Reference
1	1.68	133.0135	71.0131	C_4_H_6_O_5_	−0.75	Malic acid	[6]
2	4.95	161.0448	323.097 ([2M–H]^−^) 143.0343 ([M–H_2_O]^−^)	C_6_H_10_O_5_	−0.75	2-Hydroxyadipic acid	[7]
3	18.44	137.0237	93.0337 ([M–H–CO_2_]^−^)	C_7_H_6_O_3_	−0.72	Salicylic acid	[8,9]
4	18.81	579.1708	271.0595 ([narigenin–H]^−^)	C_27_H_32_O_14_	−1.13	Naringin	[10,11]
5	19.46	245.0919	491.1914 ([2M–H]^−^)	C_13_H_14_N_2_O_3_	−1.27	*N*-Acetyl-tryptophan	[12,13,14]
6	19.48	625.1381	300.0259 ([quercetin–H]^−^^•^) 301.0337 ([quercetin–H]^−^)	C_27_H_30_O_17_	−2.92	Quercetin-di-*O*-hexoside	[15,16]
7	20.14	609.1426	284.0310 ([kaempferol–H]^−^^•^) 285.0389 ([kaempferol–H]^−^)	C_27_H_30_O_16_	−3.51	Kaempferol-di-*O*-hexoside	[16]
8	21.17	187.0991	375.1929 ([2M–H]^−^)	C_9_H_16_O_4_	1.52	Azelaic acid	[17,18]

Rt, retention time; Exptl. [M–H]^−^, experimental *m/z* of molecular ion.

**Table 2:** Total phenolic content and free radical scavenging capacities of tomato seed flour extracts.

Sample	TPC	ORAC	DPPH	ABTS
	mg GAE/g	µmoles TE/g	µmoles TE/g	µmoles TE/g
TSF1	2.00 ± 0.11	88.57 ± 2.42	3.57 ± 0.09	3.39 ± 0.08
TSF2	1.97 ± 0.30	86.32 ± 7.01	3.81 ± 0.20	3.58 ± 0.61

TSF1, tomato seed flour extract from the first batch; TSF2, tomato seed flour extract from the second batch; TPC, total phenolic content; ORAC, oxygen radical absorbing capacity; DPPH, 2,2-diphenyl-1-picrylhydrazyl radical scavenging capacity; ABTS, 2,2′-azino-bis(3-ethylbenzothiazoline-6-sulfonic acid) cation radical scavenging capacity; GAE, gallic acid equivalents; TE, Trolox equivalents; all results are reported on a per tomato seed flour dry weight basis.

## Data Availability

The data presented in this study are available in the article.

## Supplementary Materials

The following are available online. Figure S1: Molecular structures of the compounds identified in tomato seed flour extracts, Figure S2: MTT assays of THP-1 macrophage cells using different concentrations of tomato seed flour extracts.
