# Supplementary material for: Chemical Composition of Tomato Seed Flours, and Their Radical Scavenging, Anti-Inflammatory and Gut Microbiota Modulating Properties
Source: Molecules. 2021 Mar 9;26(5):1478. doi: 10.3390/molecules26051478 (PMC7963151; doi:10.3390/molecules26051478)
Supplement: Supplementary file 1 [file molecules-26-01478-s001.pdf]

Chemical Composition of Tomato Seed Flours, and Their Radical Scavenging, Anti-inflammatory and Gut Microbiota Modulating properties

Uyory Choe [1,2], Jianghao Sun [3], Elena Bailoni [1], Pei Chen [3], Yanfang Li [1,4*], Boyan Gao [1,4], Thomas T.Y. Wang [5], Jiajia Rao [2], and Liangli (Lucy) Yu [1]

[1] Department of Nutrition and Food Science, University of Maryland, College Park, MD 20742, USA

[2] Food Ingredients and Biopolymers Laboratory, Department of Plant Sciences, North Dakota State University, Fargo, ND 58102, USA

[3] Methods and Application of Food Composition Laboratory, Beltsville Human Nutrition Research Center, Agricultural Research Service, United States Department of Agriculture, Beltsville, MD 20705, USA

[4] Institute of Food and Nutraceutical Science, School of Agriculture and Biology, Shanghai Jiao Tong University, Shanghai 200240, China

[5] Diet, Genomics and Immunology Laboratory, Beltsville Human Nutrition Research Center, Agricultural Research Service, United States Department of Agriculture, Beltsville, MD 20705, USA

* Correspondence: zoe_li@sjtu.edu.cn (Y.L.); Tel.: +86-021-34204538

Supplementary materials

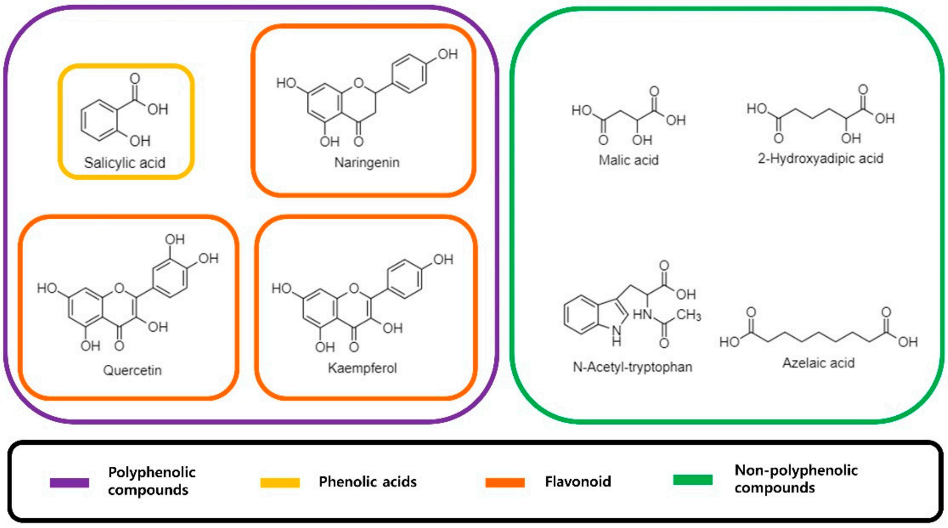

Figure S1. Molecular structures of the compounds identified in tomato seed flour extracts. Flavonoid compounds including naringin, quercetin-di-*O*-hexoside, and kaempferol-di-*O*-hexoside were shown in the form of aglycone.

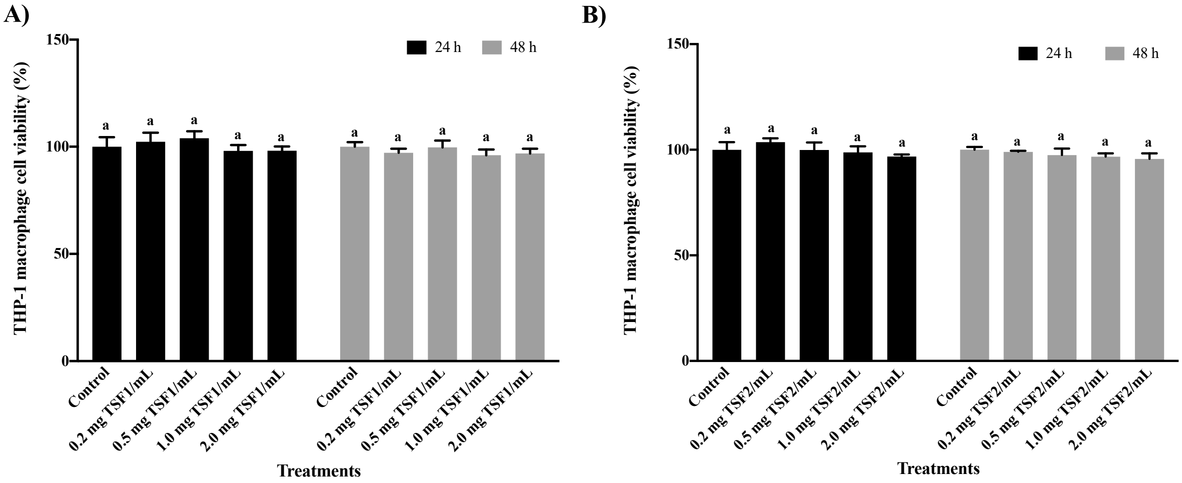

Figure S2. MTT assays of THP-1 macrophage cells using different concentrations of tomato seed flour extracts. TSF1 and TSF2 represent extracts from the first and the second batches of tomato seed flour, respectively. MTT values were normalized to the control at each time point. Different letters indicate significant difference ($P \leq 0.05$).
